# A continuous optimization approach for inferring parameters in mathematical models of regulatory networks

**DOI:** 10.1186/1471-2105-15-256

**Published:** 2014-07-29

**Authors:** Zhimin Deng, Tianhai Tian

**Affiliations:** School of Applied Mathematics, Guangdong University of Technology, Guangzhou, 510006 China; School of Mathematical Sciences, Monash University, Melbourne, 3800 Victoria Australia

**Keywords:** Parameter inference, Regulatory network, Mathematical model, Continuous objective function, Robustness analysis

## Abstract

**Background:**

The advances of systems biology have raised a large number of sophisticated mathematical models for describing the dynamic property of complex biological systems. One of the major steps in developing mathematical models is to estimate unknown parameters of the model based on experimentally measured quantities. However, experimental conditions limit the amount of data that is available for mathematical modelling. The number of unknown parameters in mathematical models may be larger than the number of observation data. The imbalance between the number of experimental data and number of unknown parameters makes reverse-engineering problems particularly challenging.

**Results:**

To address the issue of inadequate experimental data, we propose a continuous optimization approach for making reliable inference of model parameters. This approach first uses a spline interpolation to generate continuous functions of system dynamics as well as the first and second order derivatives of continuous functions. The expanded dataset is the basis to infer unknown model parameters using various continuous optimization criteria, including the error of simulation only, error of both simulation and the first derivative, or error of simulation as well as the first and second derivatives. We use three case studies to demonstrate the accuracy and reliability of the proposed new approach. Compared with the corresponding discrete criteria using experimental data at the measurement time points only, numerical results of the ERK kinase activation module show that the continuous absolute-error criteria using both function and high order derivatives generate estimates with better accuracy. This result is also supported by the second and third case studies for the G1/S transition network and the MAP kinase pathway, respectively. This suggests that the continuous absolute-error criteria lead to more accurate estimates than the corresponding discrete criteria. We also study the robustness property of these three models to examine the reliability of estimates. Simulation results show that the models with estimated parameters using continuous fitness functions have better robustness properties than those using the corresponding discrete fitness functions.

**Conclusions:**

The inference studies and robustness analysis suggest that the proposed continuous optimization criteria are effective and robust for estimating unknown parameters in mathematical models.

**Electronic supplementary material:**

The online version of this article (doi:10.1186/1471-2105-15-256) contains supplementary material, which is available to authorized users.

## Background

In systems biology, mathematical modelling plays an important role in identifying regulatory mechanisms of biochemical systems. These models have been applied successfully to study dynamic interactions among system components and simulate systems responses to external signals. Such an important tool offers enormous potential for exploring system functions at various levels and also serves as the test beds for generating hypotheses and designing biological experiments. However, one of the major challenges in mathematical modelling is the unknown model parameters that are estimated from experimentally measured quantities. Although part of the model parameters may be calculated directly from experimental data, such as the protein degradation rate that can be obtained from the half-life of the protein, the majority of model parameters has to be estimated by matching computer simulations to experimental data. In spite of substantial progresses achieved in the last 20 years, this reverse-engineering problem still remains a challenge in computational biology [1, 2]. The key issue is how to infer a large number of model parameters from a small number of experimental data [3]. Moreover, this reverse-engineering problem has been extended from parameter estimation to model selection [4, 5].

There are two major types of approaches for estimating model parameters. The first approach uses optimization techniques to find the optimal parameter sets [2]. These optimization methods usually convert parameter inference into a functional optimization problem and then minimize the objective function that measures the goodness-of-fit of the model according to the given experimental data. Two major types of optimization methods in the literature are called gradient-based optimization methods and evolutionary-based optimization methods. Based on these two basic approaches, various techniques such as simulated annealing, linear and non-linear least-squares fitting, genetic algorithms and evolutionary computation have been employed to build computational biology models [6–9]. In addition, to address the key issue of local maxima in optimization methods, deterministic and stochastic global optimization methods have been explored recently in systems biology [10].

Another type of inference methods is statistical methods including the Bayesian inference methods [11–14]. The major advantage of these methods is the ability to infer the whole probability distribution of parameters, rather than a single optimal estimate. In addition, they can deal with noisy data and uncertain data. Recent advances in Bayesian computation including Markov chain Monte Carlo (MCMC) techniques and sequential Monte Carlo (SMC) methods that have been successfully applied to infer models of biological systems [15, 16]. However, the potential obstacles of these methods in application include the potential correlation of generated samples, dependence of algorithm performance on prior hypothesis, and requirement of likelihood function. To address these issues, for example, for parameter estimation problems without analytically likelihoods, approximate Bayesian computation (ABC) methods have been designed to provide stable parameter estimates with relatively high computational efficiency [17], and thus have been applied to a wide range of models in systems biology [18]. Most recently, we have proposed two algorithms to improve the performance of ABC algorithms using the simulated likelihood density [19].

Each inference method mentioned above has its advantages and disadvantages. It is difficult to find an inference method that is effective to all the biological models and is better than all the other methods [20]. We may develop inference methods that are good enough within the given tolerances. There are a number of criteria to measure the quality of an inference method, including the efficiency regarding the computing time, flexibility about changing systems constraints, and robustness to the noise in experimental data [21]. In addition, a good inference algorithm should have less local minima in order to accelerate the convergence rate of optimization algorithms [20]. Currently the widely used objective functions are based on the least-squared error criterion or more sophisticated weighted objective functions for deterministic models [7, 9, 22] or likelihood functions for stochastic models [23, 24]. To address the issue of local maxima, a number of objective functions have been proposed using smoothness conditions of simulations such as the slope error criterion [25] and the second derivative criterion [20]. In addition, the combination of different objective functions leads to more sophisticated multiple-objective approaches that embed different sources of information into a single computational framework [26, 27].

Another important characterisitcs of inference methods is the identifiability, namely whether the model parameters can be uniquely determined by the given experimental data [2, 28]. When the number of experimental data is less than the number of unknown parameters, the inverse problem is non-identifiable. To address the issue of insufficient data, we used experimental data at measurement time points and a linear interpolation to generate more data at other time points [29]. In this work we go one step further by proposing a novel approach to infer unknown parameters in mathematical models. In addition to minimize the difference between simulation and experimental data at the measurement time points using existing methods, the innovation of this approach is to reduce potential errors at non-measurement time points. To achieve this, we propose to use a cubic spline interpolation to generate continuous representation of system dynamics and calculate simulation errors over the entire time interval of the observation. Based on the least-squared error criterion, we propose a number of continuous approaches using function values as well as the first and second order derivatives. Although the continuous representation of systems dynamics has been employed for other problems such as the continuous representation of microarray gene expression data [30], this is the first time that it is used to infer model parameters. Following this, three case studies are presented to demonstrate the effectiveness and accuracy of the proposed continuous approaches.

## Methods

### Objective functions

Here we are interested in the problem of estimating parameters in the following ODE model
1

where *X* = (*X*_1_, …, *X*_*n*_)^*T*^ are the solution of the model, and *θ* = (*θ*_1_, …, *θ*_*m*_) are the unknown model parameters. The parameter inference is searching for the optimal value of *θ* for which the simulation (*X*_*i*0_, …, *X*_*iN*_) (*i* = 1, …, *n*) of model (Eq. 1) at time points (*t*_0_, …, *t*_*N*_) have the best-fit to experimental observations (*X*_*i*_(*t*_0_), …, *X*_*i*_(*t*_*N*_)). Here *X*_*ij*_ is the simulated *i*-th component at time point *t*_*j*_, and *X*_*i*_(*t*_*j*_) the experimental data of the *i***-**th component at *t*_*j*_. A major issue in parameter inference is the criterion to measure the best-fit. The widely used criterion is the least-squared error in the form of either an absolute error  or a relative error , given by
23

In addition to the error of function values, the slope error criterion has been introduced to infer parameters in the S-system model [31], which is an ODE model to describe the dynamics of metabolic networks. The absolute and relative slope errors are given by
45

where  denotes the slope of experimental data for the *i*-th component at time point *t*_*j*_, and  is the numerical derivative of the *i*-th simulated component at *t*_*j*_.

Similarly, the criteria based on the second derivative are introduced here as
6789

where *X*′′ _*i*_(*t*_*j*_) is the second derivative of *X*_*i*_(*t*_*j*_). These criteria are designed either to reduce the errors of the second order derivatives (6, 7) or to decrease the “roughness” of the solution, and hence to relieve the overfitting problem (8, 9) [20].

In addition, there are potentially some large simulation errors at the non-measurement time points even when the simulation is in good agreement with experimental data at the measurement time points. To improve the reliability of inference, one approach is to generate observation data at a number of non-measurement time points by using a linear interpolation [29]. However, such a low order interpolation may generate errors to the generated observation data and further influence the accuracy of inferred model parameters. To improve the accuracy, this technique can be extended in one of the following two ways: we may either use a higher order interpolation method or estimate observation data at more non-measurement time points. The extreme case of the latter approach is to use the whole trajectory rather than observations at discrete time points to calculate the difference between simulated and experimental data. Here we use this approach to develop a continuous optimization method to infer model parameters reliably.

We first expand the discrete experimental data into the continuous function *X*_*i*_(*t*) (*t* ∈ [*a*, *b*]) by using a cubic spline interpolation. After obtaining numerical solution  of the model (Eq. 1) in the same interval, we define the following continuous optimization criteria, given by
1011121314151617

To take full advantage of each criterion, we develop a number of multiple-objective fitness functions to estimate model parameters, which are listed in Table 1. Note that the integrals in the above criteria are calculated using a numerical integration method such as the composite Simpson’s rule. The maximal value of a function over the interval [a, b] is defined as the maximum of function values at the sampling points.

**Table 1:** **Fitness functions for measuring simulation error**

Criterion	Notation	Definition	Comment
DAE1		Eq. (2)	Discrete absolute error of function values
DAE2		(2, 4)	Discrete absolute error of function and derivative values
DAE3		(2, 4, 6)	Discrete absolute error of function, derivative, second derivative values
DAE4		(2, 4, 8)	Discrete absolute error of function, derivative values as well as values of second derivative.
DRE1		(3)	Discrete relative error of function values
DRE2		(3, 5)	Discrete relative error of function and derivative values
DRE3		(3, 5, 7)	Discrete relative error of function, derivative, second derivative values
DRE4		(3, 5, 9)	Discrete relative error of function, derivative values as well as values of second derivative.
CAE1		(10)	Continuous absolute error of function values
CAE2		(10, 12)	Continuous absolute error of function and derivative values
CAE3		(10, 12, 14)	Continuous absolute error of function, derivative, second derivative values
CAE4		(10, 12, 16)	Continuous absolute error of function, derivative values as well as values of second derivative.
CRE1		(11)	Continuous relative error of function values
CRE2		(11, 13)	Continuous relative error of function and derivative values
CRE3		(11, 13, 15)	Continuous relative error of function, derivative, second derivative values
CRE4		(11, 13, 17)	Continuous relative error of function, derivative values as well as values of second derivative.

(DAE: discrete absoulte error, CAE: continuous absolute error, DRE: discrete relative error, CRE: continuous relative error).

### Cubic spline interpolation

The essential idea of the cubic spline is to fit data by using a piecewise function of the form


where each *t*_*i*_ is the time point of observed data, and *s*_*i*_(*t*) is a cubic polynomial, defined by


for *i* = 1, …, *m* - 1. The cubic spline S(t) will interpolate all data points. In addition, the function, first and second derivatives are continuous for the interval [*t*_1_, *t*_*m*_]. Compared with the piece-wise linear interpolation, a cubic spline function has two major advantages: a higher order of approximation to increase approximation accuracy and smoothness of first and second derivatives. The latter is important in this work since we include the first and second derivatives of simulation/observation to measure simulation errors.

There are various types of the cubic spline functions depending on different boundary conditions. Denoting the second derivative of the spline as *M*_*i*_ = *S*′′ (*t*_*i*_), three examples of cubic spline interpolation are natural spline (*M*_1_ = *M*_*m*_ = 0), parabolic spline (*M*_1_ = *M*_2_, *M*_*m* - 1_ = *M*_*m*_) and cubic runout spline (*M*_1_ = 2*M*_2_ - *M*_3_, *M*_*m*_ = 2*M*_*m* - 1_ - *M*_*m* - 2_). This work uses the natural spline interpolation to generate continuous functions for numerical solutions of system (Eq. 1) and for experimental data.

### Genetic algorithm

All model parameters are estimated using a genetic algorithm, which is an effective searching method for finding unknown kinetic rates when the search space is associated with a complex error landscape. We use a MATLAB toolbox developed by Chipperfield et al. [32] to infer the unknown model parameters. This toolbox uses MATLAB functions to build a set of versatile routines for implementing a wide range of genetic algorithms. The major procedures of the genetic algorithm toolbox include population representation and initiation, fitness assignment, selection functions, crossover operators, mutation operators and multiple subpopulation support. In this work we use the function *crtbp* to create the binary initial population. The linear-ranking and non-linear-ranking algorithm *ranking* is used to transform the raw objective function values into non-negative figures of merit for each individual. In addition, a selection function *reins* is used to effect fitness-based reinsertion when the entire population is not reproduced in each generation, and a high-level entry function *select* is used to provide a convenient interface to the selection routines. Finally, a high-level entry function *recombine* and the routine *mut* are applied to provide all the crossover operators and perform binary and integer mutations.

In our numerical tests, the genetic algorithm run over 300 generations for each estimate and there are a population of 100 individuals in each generation. The estimation error generally remains unchanged after the 200th generation in each implementation. The value of a model parameter is taken initially from the uniform distribution in the range of [0,W_max_]. Here W_max_ is the maximal possible value of that parameter. Different parameters may have different values of W_max_. The initial estimate of rate constants can be changed by using different random seeds in the MATLAB toolbox, leading to different final estimates of the model parameters. We use different seeds of random numbers in MATLAB to generate different initial sets of model parameters in the genetic algorithm. For each initial set of parameters, we simulate the mathematical model to obtain the time-course profiles of the system. Different criteria listed in Table 1 are used in the genetic algorithm as the objective function to calculate the difference between numerical and standard simulations. For the discrete criteria, we simply compare the differences between the simulated and exact data at each measurement time point. However, when using the continuous criteria, we use a cubic spline to obtain the continuous function as well as its first and second derivatives for both experimental data and numerical solutions. The calculated fitness value is then returned to genetic algorithm for selecting the optimal model parameters.

### Accuracy of the estimated model parameters

In this work we first use a given set of model parameters *θ* * = (*θ*_1_ *, …, *θ*_*m*_ *) to generate a simulation that is used as the observation data, and then infer the model parameters based on the generated observation data. Due to the local optimization issue for genetic algorithm, we infer a number of sets of model parameters using each continuous or discrete criterion and choose 10 sets with the smallest values of objective function for each criterion. The estimation error of each set of inferred model parameters *θ*^(*j*)^ = (*θ*_1_^(*j*)^, …, *θ*_*m*_^(*j*)^) is defined as the relative difference from the model parameters *θ* * = (*θ*_1_ *, …, *θ*_*m*_ *). The accuracy of each criterion is defined as the mean of the estimation errors, given by
18

To treat each parameter equally, we choose relative errors to measure the difference between the estimated parameters and model parameters. This mean error is a measure for accuracy property of each criterion. In addition, we present standard deviation (STD) for these 10 sets of estimates as additional information for the quality of estimates. However, the comparison of discrete and continuous approaches is mainly based on the magnitude of the mean errors of these approaches.

### Robustness analysis

The robustness property of a mathematical model with respect to a set of perturbations *P* is defined as the average of an evaluation function  of the system over all perturbations *p* ∈ *P*, weighted by the perturbation probabilities *prob*(*p*), given by [33]
19

Here we use the following measure to evaluate the robustness property


where  is the numerical solution of the i-th component at time point *t*_*j*_ using the perturbed parameters, and *x*_*ij*_ is the corresponding simulated data without parameter perturbation. In addition, the standard deviation of simulation errors is used to measure the fluctuations of perturbed simulations.

For each estimated model parameter *θ*_*i*_, the perturbation is set to
20

with the Gaussian random variable *N(0,1)*. Here *μ* = 0.2 is the perturbation strength for the first and second systems and *μ* = 0.1 for the third system. We have also tested other values of strength. When the value of *μ* is small, the difference between the robustness properties is too small to distinguish for different methods. However, if this value is large, the model with perturbed parameters may be stiff and numerical solutions may blow out.

For each set of estimated parameters, we generate 5000 sets of perturbed model parameters. The variation of each set of perturbed parameters to the unperturbed model parameters is measured by


Then we calculate the mean and standard deviation (STD) of the variance *ϵ*_*l*_. For each criterion, we considere the top 10 sets of estimated parameters that have the smallest values of the fitness function. The mean and STD of the variation *ϵ*_*l*_ for each set of parameter are averaged, which are used as the final results presented in Additional file 1: Tables S6, S7 and S8.

## Results

### The ERK kinase activation module

The first case study estimates the rate constants of ERK kinase activation module using experimental data [34]. The MAP kinase pathway comprises of a set of three protein kinases, namely Raf, MEK and ERK. Raf kinase is activated by the input signal Ras protein, and then these three kinases are activated sequentially [35]. In *vivo*, activated MEK activates ERK kinase dominantly in the cytosol by phosphorylating threonine and tyrosine residues in the activation loop [36]. In the process of distributive catalysis, the activated MEKpp that binds to the substrate ERK, activates one of the sites and releases the intermediate mono-phosphorylated ERKp. Then, a new collision between MEKpp and ERKp is required for the conversion of this intermediate into the dual-phosphorylated ERKpp. The biochemical reactions for the ERK activation are represented by


Based on the above reactions, the mathematical model for a system of six differential equations is given by
21

where [M] and [E] are the concentrations of MEK and ERK, respectively, and [Mpp-E] represents complex [MEKpp-ERK].

We use a stiff-implicit solver *ode23tb* in MATLAB to simulate model (Eq. 21) with rate constants [37]
22

and initial condition [0.165, 5, 0, 0, 0, 0] (in Figure three in [34]). The generated simulation is used as the standard simulation which is very close to the experimental data (Additional file 1: Figure S1). The natural cubic spline is used to calculate the first and second derivatives of the standard simulation. Then we use a genetic algorithm to search for the optimal model parameters [32]. The values of W_max_ for these 6 parameters all are equal to 300.

**Figure 1:**
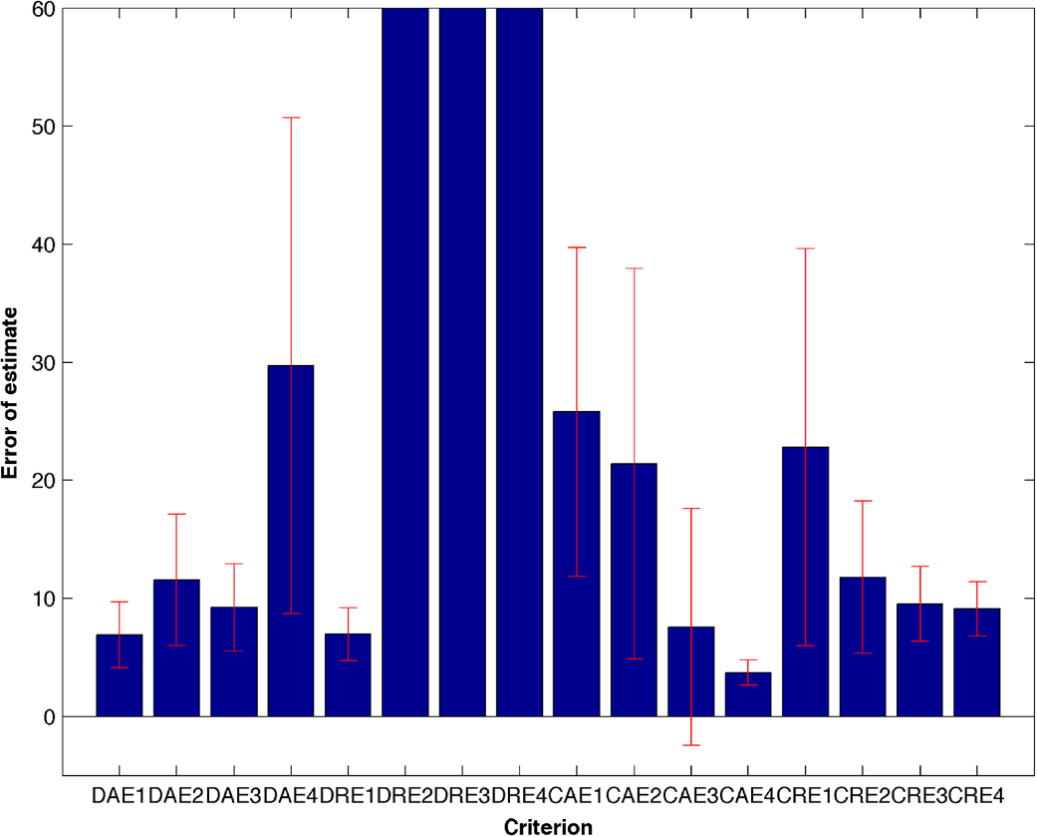
**Mean error and STD of different approaches for imferring the ERK kinase activation module.** Criterion CAE4 has the smallest values of both mean error and STD.

For each criterion in Table 1 we estimate 100 sets of parameters and selected the top 10 sets that have the smallest fitness values. Additional file 1: Figure S1 shows that the standard simulation is very close to the simulation using the estimated model parameters. Since the fitness functions are based on different criteria, it is not appropriate to compare fitness values directly. Thus we calculated the mean error (Eq. 18) and STD of the estimated parameter sets, which are presented in Figure 1 and Additional file 1: Table S2. Numerical results in Table 2 and Additional file 1: Table S2 suggest that the continuous criteria with function values as well as the first and second derivatives (CAE4) give estimates with the best accuracy in terms of mean error and the most reliability in terms of the STD. In addition, the discrete criteria may generate more accurate estimates than the corresponding continuous criteria if only the function values are used to calculate the fitness values (e.g. DAE1 and DRE1). However, for the continuous criteria, more constraints based on the values of derivatives lead to more accurate estimations, which is consistent with the previous research results [20]. Furthermore, the STD of each criterion is consistent with the corresponding mean error, which suggests that the estimates of a criterion would be more stable if the estimation error is smaller.

**Table 2:** **Summary of the accuracy of the estimated model parameters**

	ERK kinase module	G1/S transition module	MAP kinase pathway
The number of the absolute criteria	4	4	0
The number of the relative criteria	4	4	4
Better continuous absolute criteria	CAE3, CAE4	CAE1, CAE2, CAE3, CAE4	N/A
Better discrete absolute criteria	DAE1, DAE2	0	N/A
Better continuous relative criteria	CRE2, CRE3, CRE4	CRE4	CRE1, CRE2, CRE3, CRE4
Better discrete relative criteria	DRE1	DRE1, DRE2, DRE3	0
The best criteria	CAE4	CAE1	CRE2

The comparison of discrete and continuous approaches is mainly based on the magnitude of the mean errors of these approaches which are given in Additional file 1: Tables S2, S3 and S4.

To find out the reasons for variations in the performance of various criteria, we plot the mean of each estimated model parameter, which is the average of the top 10 estimates for each criterion in Additional file 1: Figure S2. To match the relative errors in Additional file 1: Table S2, the averaged parameters were scaled by the exact parameter; and the exact parameters in Additional file 1: Figure S2 are always unit one. Additional file 1: Figure S2 suggests that the criterion CAE4 (the continuous absolute-error criterion using the first and second derivatives) produces estimates in which each estimated parameter is close to the exact one with moderate errors. However, the discrete relative-error criteria may produce estimates with large errors.

### The G1/S transition module

The second case study discusses an autocatalytic system of the G1/S transition module. In this network pRB (retinoblastoma) is a tumour suppressor from the family of pocket proteins, and E2F1 is a transcription factor targeting gene that regulates cell cycle progression. The tumour suppressor pRB was originally discovered in childhood cancer of the retina and turned out to be the crucial inhibitor for the G1/S phase progression. For these reasons, the pair E2F/DP (i.e. the complex of protein E2F and DP) and pRB have been regarded as the central players of the transition phenomena. Here E2F1 is its own transcriptional activator, and it is also a transcription factor for its inhibitor pRB [38]. This module of two genes shows bistability dynamics, which is described by the following equations [38].
23

For the 10 parameters in the model, we use the exact values (*k*_1_ = 1, *K*_*n*1_ = 0.5, *J*_11_ = 0.5, *ϕ*_*pRB*_ = 0.005, *k*_*p*_ = 0.05, *k*_2_ = 1.6, *a* = 0.04, *K*_*n*2_ = 4, *J*_12_ = 5, *ϕ*_*E*2*F*1_ = 0.1) and initial conditions [pRB, E2F1] = [1,5] [38] to generate a standard simulation whose values at t = [0, 50, 100, 150, 200, 250, 300] are used as the experimental data (Additional file 1: Figure S3).

We employed the same approach as used for the first case study to investigate the accuracy of different criteria. The values of W_max_ for these 10 parameters all are equal to 5. Numerical results in Table 2 and Additional file 1: Table S3 suggested that, among the absolute-error criteria, the continuous criteria always generate more accurate estimates than the corresponding discrete criteria. In addition, among all types of continuous criteria, the absolute-error criteria produced more accurate estimates than the relative-error criteria. Therefore, continuous absolute-error criteria are superior to the other criteria in both mean error and STD. In particular, continuous absolute-error criterion of function values (CAE1) has the minimal mean error. Numerical results in the second case study suggested that the continuous absolute-error criteria are effective approaches for inferring unknown parameters in mathematical models.

### The MAP kinase pathway

The first two tested models have only small numbers of unknown parameters. Therefore the next question is whether the results obtained from these two small-scale systems are still valid for systems with a large number of unknown parameters. To answer this question, we used a recently proposed model with 57 unknown model parameters as the third test system. This model describes the dynamics of the MAP kinase pathway, which is one of the most prominent signaling pathways [29]. This model comprises a cytosolic subsystem and a nuclear subsystem. In the cytosolic subsystem, the Ras-GTP is the signal input of the MAP kinase cascade, which activates Raf molecules in a single step. This activation is followed by sequential activation of the dual-specificity MAP kinase kinase (MEK) by Raf* (i.e., the activated Raf) in a single-step processive module. The activated MEKpp in turn activates ERK in a two-step distributive module. The activated ERKpp is the signal output of the MAK kinase module [29, 36]. MEK and ERK kinases can diffuse between the cytosol and nucleus freely. In the nuclear subsystem, the activated MEKpp can further activate ERK kinase via the distributive two-step phosphorylation module. In addition, phosphatases, termed as Raf-P’ase, MEK-P’ase and ERK-P’ase, can deactivate the activated Raf*, MEKpp and ERKpp kinases, respectively, at different subcellular locations [29]. The detailed information of phosphorylation and dephosphorylation reactions as well as the differential equation model are given in the Additional file 1.

The extended observation data, namely the proteomic data together with the interpolated data at the time points between the observations, are used to infer the model parameters. The proteomic data are relative protein concentrations, in which the activity of each kinase is normalized by its activity at 5 min. Thus we only use the four relative criteria (DRE1 ~ 4 and CRE1 ~ 4) to infer the model parameters. We also use the kinetic rate constants estimated from the same dataset [29] to generate a simulation. The rate constants and initial conditions are also provided in the Additional file 1. Additional file 1: Figure S4 gives simulated and observed datasets. Similar to the experimental data, we use the activities of Raf, MEK and ERK kinases to infer parameters. Here MAK and ERK data include the activities in cytosol, nucleus as well as the total activity. We use a stiff-implicit solver *ode23tb* in MATLAB to simulate mathematical model and apply genetic algorithm to search for optimal model parameters [32]. The cubic spline interpolation is used to calculate the first and second derivatives of the observation data and numerical simulation. The values of W_max_ for these 57 unknown parameters all are equal to 300.

For each relative criterion, we estimate 100 sets of parameters and selected the top 10 sets that have the smallest fitness values. Then we compared the relative difference between the estimated parameters and exact rate constants, which were presented in Additional file 1: Table S4. For various fitness functions, the continuous criteria always generated more accurate estimates than the discrete criteria. In particular, among various continuous criteria, the criterion based on the simulation values (CRE1) leads to estimates with the smallest mean error; in addition, the criterion using function values and the first order derivative values (CRE2) produced more stable estimates, which have smaller values of the STD.

For the process of kinase activation, it was indicated that the steady state levels of the kinases were determined by the factor
24

rather than by the individual rate constants that are pertinent [39]. Thus we calculated the averaged errors of the factor *K*_*mi*_ for each inference method we have tested, which are presented in Additional file 1: Table S5. Numerical results suggest that continuous criterion using the function values (CRE1) generates estimates with the best accuracy and smallest STD.

### Robustness analysis

Due to the lack of experimental data as a constraint in reverse-engineering, the estimated model parameters may have a wide range of values but all of them are able to faithfully realize experimental observations. Currently a few additional criteria, including robustness property, have been used to select the candidates of estimated parameters. Robustness can be defined as the ability of a system to function correctly in the presence of both internal and external uncertainty [40]. Since the robustness property is ubiquitously in biological systems [41, 42], it has been used as a criterion not only to select the optimal network from the candidate structures but also to choose estimated rate constants of mathematical models [43–45]. A formal definition of this property is well consistent with the general principle of robustness property for complex systems [33, 40]. Recently more detailed definitions have been proposed to calculate the robustness property of biological systems [46].

To demonstrate the reliability of the continuous approach, we then carry out the robustness analysis for the three systems with the selected top 10 estimates of model parameters of each criterion. Here noise in the model represents the combined sources of fluctuations such as errors in estimated parameters, external environmental noise and internal noise due to small numbers of molecules. We first used the estimated kinetic rates without any perturbation to produce a standard simulation. Then for each set of parameters, we generated 5,000 sets of perturbed rate constants using the Gaussian random variable and a perturbation strength *μ* (Eq. 20). Perturbed simulations were obtained by using the perturbed parameters, and we compared the standard simulation with perturbed simulations. Based on the definition of robustness (Eq. 19), we used the mean and STD of simulation errors to represent the robustness property. The system with a particular set of rate constants is more stable if both the mean and STD of simulation errors are smaller.

For the first model of ERK kinase activation, Table 3 and Additional file 1: Table S6 shows that the models derived from the continuous absolute-error criterion have slightly better robustness properties than those derived from the continuous relative-error criterion or discrete absolute-error criterion. They also show much better robustness properties than those derived from the discrete relative-error criterion. However, for the second model of the G1/S transition module, the robustness properties of the continuous approaches in Table 3 and Additional file 1: Table S7 are merely as stable as those for discrete approaches. All the mean fitness values and STDs of fitness values of different criteria are close to each other. In this case only the estimates derived from the continuous absolute-error criteria have better robustness properties than those derived from the corresponding discrete criteria. For the MAP kinase pathway, robustness analysis results in Table 3 and Additional file 1: Table S8 suggest that the generated estimates from three continuous approaches out of four have better robustness property than those from the discrete approaches. Thus the robustness analysis for those three test systems shows that two models with the parameters generated using continuous criteria create better robustness properties than the corresponding models derived from discrete criteria, while the remaining model with parameters from either continuous approaches or discrete approaches has similar robustness properties. In summary, robustness analysis results suggest that the continuous approaches have generated estimates of model parameters that are either more stable than or as stable as those derived from the discrete approaches.

**Table 3:** **Summary of the robustness property of the three models with estimated model parameters**

	ERK kinase module	G1/S transition module	MAP kinase pathway
The number of the absolute criteria	4	4	0
The number of the relative criteria	4	4	4
Better continuous absolute criteria	CAE1, CAE3, CAE4	CAE1, CAE3	N/A
Better discrete absolute criteria	0	DAE2, DAE4	N/A
Better continuous relative criteria	CRE2, CRE3, CRE4	CRE4	CRE1, CRE2, CRE3
Better discrete relative criteria	DRE1	DRE1, DRE2, DRE3	DER4

The comparison of discrete and continuous approaches is mainly based on the magnitude of the mean errors of these approaches which are given in Additional file 1: Tables S6, S7 and S8.

## Discussion

This work presents a continuous optimization approach to address the issue of inadequate experimental data for inferring unknown parameters in mathematical models of biochemical networks. The proposed method is based on the approximation of a cubic spline function to the underlying solution of the network model. In addition to the function values that are widely used in the existing approaches, both the first and second derivatives of the simulation are also required to match the generated observation data using a cubic spline function. Therefore the success of this approach depends on the approximation of the cubic spline to the solution of network model. Another important factor to determine the success of continuous approaches may be the relative amount of added data using continuous approaches. Compared with the experimental observation data, the second model (i.e. the G1/S transition module) has less generated observation data using the continuous approaches; while the third model (i.e. the MAP kinase pathway) has much more generated observation data. The performance of continuous approaches for the third model is better than the corresponding discrete approaches. However, using continuous approaches for the second model is just as good as discrete approaches. Thus our results suggest that the performance of these continuous methods will be better if continuous approaches can generate more observation data.

For the three test models, numerical simulations using estimated model parameters match the continuous system dynamics based on experimental data very well. However, numerical results suggest that the first derivative of the system dynamics contributes the majority of simulation error. Thus new methods are needed to reduce the approximation error of the first derivative. This observation may give an explanation for the small difference between criteria 3 and 4, namely the criterion using the error of the second derivative and that using the maximal value of the second derivative. However, it will be interesting to examine the importance of higher order derivatives and consider other types of approximation methods in order to consider approximations using higher order derivatives.

It is assumed in this work that there is no noise in experimental measurement. However, the datasets from biological experiments are often subject to environmental fluctuations and measurement error. Noise at one measurement point will be propagated to the function values at other time points via interpolation function. Thus it may have substantial impact on the accuracy of estimated parameters. Since spline is a piecewise continuous interpolation, our approach can ensure that the influence of noise is constrained to a relatively small time period. Recently a number of approaches have been proposed to filter out noise from experimental observations, including the particle filtering method [47]. Thus an interesting topic is to combine these noise-filtering methods with the continuous optimization approach to make reliable estimation of unknown parameters.

The calculation of simulation error is also an important issue in parameter estimation. This work considered two types of error, namely absolute error and relative error. An interesting result is that using criteria based on absolute error lead to estimates with better accuracy, though it was widely accepted that the advantage of using relative error is to avoid the dominance of large errors derived from parameters with large value. When all the parameter values are moderate in the second test system, there is no substantial difference between the estimates derived from absolute error or relative error. Regarding the calculation of simulation error, a more sophisticated approach is using the weighted distance measure [9], which would be a topic of interest in further study.

Our proposed continuous approach represents a new starting point to design inference methods to estimated unknown parameters in mathematical models. Nearly all current inference methods are discrete methods using experimental data at a number of measurement time points. Thus we may design corresponding continuous approaches based on these discrete approaches. The key issue is whether the continuous inference method is better than the corresponding discrete method in terms of accuracy and reliability. In this work we compared the accuracy of four discrete inference methods with that of the corresponding continuous methods. In fact these discrete methods are current in use for estimating model parameters [20, 29, 31]. Thus this work provided promising evidence showing that the continuous approaches perform better than the corresponding discrete methods for the three test systems.

## Conclusions

In summary, this work proposes an effective continuous optimization approach for estimating model parameters in mathematical models of biological systems. This method uses a spline function to approximate the underlying solution of the network model. The spline function generates observation data at other time points together with the first and second derivatives of the network simulation. All the generated information is used to infer unknown parameters in mathematical models. This work represents an early attempt to address the issue of inadequate experimental data for estimating unknown parameters. Three case studies are used to verify the accuracy of the proposed approaches. Simulation results suggest that the proposed continuous optimization method is an effective and robust approach for accurate and reliable parameter estimation.

## Electronic supplementary material

Additional file 1: **Provides the ODE model and rate constants for the MAP kinase pathway.** It also gives detailed information regarding the estimation error and robustness property of various methods for the three test systems. Supplementary Figures show the simulated network dynamics and estimated values of each unknown parameter. (PDF 1 MB)
