# Supplementary material for: A continuous optimization approach for inferring parameters in mathematical models of regulatory networks
Source: BMC Bioinformatics. 2014 Jul 29;15(1):256. doi: 10.1186/1471-2105-15-256 (PMC4261783; doi:10.1186/1471-2105-15-256)
Supplement: Supplementary file 1 — Additional file 1: Provides the ODE model and rate constants for the MAP kinase pathway. It also gives detailed information regarding the estimation error and robustness property of various methods for the three test systems. Supplementary Figures show the simulated network dynamics and estimated values of each unknown parameter. (PDF 1 MB) [file 12859_2013_6685_MOESM1_ESM.pdf]

Supplementary Information

A continuous optimization approach for inferring parameters in mathematical models of regulatory networks

Zhimin Deng and Tianhai Tian

This supplementary information gives the ODE model and rate constants for the third test system: the MAP kinase pathway. The information directly comes from reference [1]

$$\frac{d[Ras]}{dt} = -a_1[Ras][Raf] + (d_1 + k_1)[Ras - Raf]$$

$$\frac{d[Raf]}{dt} = -a_1[Ras][Raf] + d_1[Ras - Raf] + k_2[Raf* - RafP'ase]$$

$$\frac{d[Ras - Raf]}{dt} = a_1[Ras][Raf] - (d_1 + k_1)[Ras - Raf]$$

$$\frac{d[Raf*]}{dt} = -a_2[Raf*][RafP'ase] + d_2[Raf* - RafP'ase] + k_1[Ras - Raf] - a_3[Raf*][MEK] + (d_3 + k_3)[Raf* - MEK]$$

$$\frac{d[RafPase]}{dt} = -a_2[Raf*][RafP'ase] + (d_2 + k_2)[Raf* - RafP'ase]$$

$$\frac{d[Raf* - RafPase]}{dt} = a_2[Raf*][RafP'ase] - (d_2 + k_2)[Raf* - RafP'ase]$$

$$\frac{d[MEK]}{dt} = -a_3[Raf*][MEK] + d_3[Raf* - MEK] + k_4[MEKp - MEKPase] + f_3[N - MEK] - f_7[MEK]$$

$$\frac{d[Raf* - MEK]}{dt} = a_3[Raf*][MEK] - d_3[Raf* - MEK]$$

$$\frac{d[MEKp]}{dt} = -a_4[MEKp][MEKPase] + d_4[MEKp - MEKP'ase] + k_5[MEKpp - MEKPase] - f_9[N - MEKp] + f_{10}[MEKp]$$

$$\frac{d[MEKpp]}{dt} = -a_5[MEKpp][MEKPase] + d_5[MEKpp - MEKP'ase] + k_3[Raf* - MEK] - a_6[MEKpp][ERK] + (d_6 + k_6)[MEKpp - ERK] - a_7[MEKpp][ERKp] + (d_7 + k_7)[MEKpp - ERKp] - f_1[MEKpp] + f_5[N - MEKpp]$$

$$\frac{d[MEKP'ase]}{dt} = -a_4[MEKp][MEKPase] + (d_4 + k_4)[MEKp - MEKP'ase] - a_5[MEKpp][MEKPase] + (d_5 + k_5)[MEKpp - MEKP'ase] - a_{10}[N - MEKp][MEKPase] + (d_{10} + k_{10})[N - MEKp - MEKP'ase] - a_{11}[N - MEKpp][MEKPase] + (d_{11} + k_{11})[N - MEKpp - MEKP'ase]$$

$$\frac{d[MEKp - MEKP'ase]}{dt} = a_4[MEKp][MEKPase] - (d_4 + k_4)[MEKp - MEKP'ase]$$

$$\frac{d[MEKpp - MEKP'ase]}{dt} = -a_5[MEKpp][MEKPase] + (d_5 + k_5)[MEKpp - MEKP'ase]$$

$$\frac{d[ERK]}{dt} = -a_6[MEKpp][ERK] + d_6[MEKpp - ERK] + k_8[ERKp - ERKPase] + f_4[N - ERK] - f_8[ERK]$$

$$\frac{d[MEKpp - ERK]}{dt} = a_6[MEKpp][ERK] - (d_6 + k_6)[MEKpp - ERK]$$

$$\frac{d[ERKp]}{dt} = -a_7[MEKpp][ERKp] + d_7[MEKpp - ERKp] + k_6[MEKpp - ERK] + f_{12}[N - ERKp] - f_{11}[ERKp]$$
$$- a_8[ERKp][ERKP'ase] + d_8[ERKp - ERKP'ase] + k_7[ERKpp - ERKP'ase]$$
$$\frac{d[MEKpp - ERKp]}{dt} = a_7[MEKpp][ERKp] - (d_7 + k_7)[MEKpp - ERKp]$$
$$\frac{d[ERKpp]}{dt} = -a_9[ERKpp][ERKPase] + d_9[ERKpp - ERKP'ase] + k_7[MEKpp - ERKp]$$
$$- f_2[ERKpp] + f_6[N - ERKpp]$$
$$\frac{d[ERKP'ase]}{dt} = -a_8[ERKp][ERKPase] + (d_8 + k_8)[ERKp - ERKP'ase]$$
$$- a_9[ERKpp][ERKPase] + (d_9 + k_9)[ERKpp - ERKP'ase]$$
$$- a_{12}[N - ERKp][ERKPase] + (d_{12} + k_{12})[N - ERKp - ERKP'ase]$$
$$- a_{13}[N - ERKpp][ERKPase] + (d_{13} + k_{13})[N - ERKpp - ERKP'ase]$$
$$\frac{d[ERKp - ERKP'ase]}{dt} = a_8[ERKp][ERKPase] - (d_8 + k_8)[ERKp - ERKP'ase]$$
$$\frac{d[ERKpp - ERKP'ase]}{dt} = -a_9[ERKpp][ERKPase] + (d_9 + k_9)[ERKpp - ERKP'ase]$$
$$\frac{d[N - MEK]}{dt} = k_{10}[N - MEKp - MEKP'ase] - f_3[N - MEK] + f_7[MEK]$$
$$\frac{d[N - MEKp]}{dt} = -a_{10}[N - MEKp][MEKP'ase] + d_{10}[N - MEKp - MEKPase]$$
$$+ k_{11}[N - MEKpp - MEKP'ase] - f_3[N - MEKp] + f_7[MEKp]$$
$$\frac{d[N - MEKp - MEKP'ase]}{dt} = a_{10}[N - MEKp][MEKP'ase] - (d_{10} + k_{10})[N - MEKp - MEKPase]$$
$$\frac{d[N - MEKpp]}{dt} = -a_{11}[N - MEKpp][MEKP'ase] + d_{11}[N - MEKpp - MEKPase]$$
$$- f_5[N - MEKpp] + f_1[MEKpp]$$
$$\frac{d[N - MEKpp - MEKP'ase]}{dt} = a_{11}[N - MEKpp][MEKP'ase] - (d_{11} + k_{11})[N - MEKpp - MEKPase]$$
$$\frac{d[N - ERK]}{dt} = -a_{14}[N - MEKpp][N - ERK] + d_{14}[N - MEKpp - N - ERK]$$
$$+ k_{12}[N - ERKp - MEKP'ase] - f_4[N - ERK] + f_8[ERK]$$
$$\frac{d[N - ERKp]}{dt} = -a_{12}[N - ERKp][ERKP'ase] + d_{12}[N - ERKp - ERKP'ase]$$
$$+ k_{13}[N - ERKpp - MEKP'ase] - f_2[N - ERKp] + f_{11}[ERKp]$$
$$- a_{15}[N - MEKpp][N - ERKp] + d_{15}[N - MEKpp - N - ERKp] + k_{14}[N - MEKpp - N - ERK]$$
$$\frac{d[N - ERKp - ERKP'ase]}{dt} = a_{12}[N - ERKp][ERKP'ase] - (d_{12} + k_{12})[N - ERKp - ERKP'ase]$$
$$\frac{d[N - ERKpp]}{dt} = -a_{13}[N - ERKpp][ERKP'ase] + d_{13}[N - ERKpp - ERKP'ase]$$
$$+ k_{15}[N - MEKpp - N - ERKp] + f_2[ERKpp] - f_6[N - ERKpp]$$
$$\frac{d[N - ERKpp - ERKP'ase]}{dt} = a_{13}[N - ERKpp][ERKP'ase] + (d_{13} + k_{13})[N - ERKpp - ERKP'ase]$$
$$\frac{d[N - MEKpp - N - ERK]}{dt} = a_{14}[N - MEKpp][N - ERK] + (d_{14} + k_{14})[N - MEKpp - N - ERK]$$
$$\frac{d[N - MEKpp - N - ERKp]}{dt} = a_{15}[N - MEKpp][N - ERKp] + (d_{15} + k_{15})[N - MEKpp - N - ERKp]$$

Supplementary Table 1: Model kinetic rates

a1	12.9004
a2	296.9649
a3	787.5564
a4	962.5454
a5	117.0922
a6	8.1720
a7	110.5682
a8	22.9655
a9	50.1080
a10	413.9485
a11	578.0903
a12	545.6148
a13	597.8275
a14	715.5311
a15	950.0722
d1	829.0270
d2	269.5801
d3	931.6711
d4	411.4126
d5	461.8659
d6	992.0931
d7	941.7581
d8	542.6078
d9	985.8656
d10	228.2631
d11	683.4208
d12	359.6071
d13	984.9748
d14	310.1609
d15	876.6049
k1	673.1917
k2	347.7281
k3	527.5817
k4	94.4081
k5	966.8493
k6	905.9800
k6	683.1633
k7	860.5174
k9	776.3679
k10	150.1161
k11	486.3171
l12	415.2683
k13	17.8394
k14	625.4545
k15	450.5782
f1	856.2792

f2	981.8249
f3	549.4905
f4	97.4713
f5	5.0669
f6	146.6915
f7	34.3962
f8	858.4803
f9	897.4122
f10	453.4173
f11	951.8842
f12	7.1974

Supplementary Table 2 - Averaged errors of the estimated model parameters for the model of the ERK kinase activation module (STD: standard deviation)

Criterion	Discrete criterion		Criterion	Continuous criterion	
	Mean error	STD		Mean error	STD
DAE1	6.89	2.79	CAE1	25.81	13.93
DAE2	11.56	5.55	CAE2	21.40	16.56
DAE3	9.23	3.70	CAE3	7.58	10.04
DAE4	29.73	21.00	CAE4	<u>3.72</u>	<u>1.06</u>
DRE1	6.98	2.24	CRE1	22.80	16.85
DRE2	510.96	0.26	CRE2	11.78	6.45
DRE3	208.41	34.28	CRE3	9.53	3.18
DRE4	257.81	17.77	CRE4	9.11	2.29

Supplementary Table 3 - Averaged errors of the estimated model parameters for model of the G1/S transition module.

Criterion	Discrete criterion		Criterion	Continuous criterion	
	Mean error	STD		Mean error	STD
DAE1	7.40	2.48	CAE1	<u>5.33</u>	1.98
DAE2	7.24	2.09	CAE2	5.89	1.72
DAE3	7.11	1.91	CAE3	5.89	1.72
DAE4	6.82	2.03	CAE4	5.99	1.58
DRE1	6.54	1.17	CRE1	6.77	2.38
DRE2	6.71	2.28	CRE2	7.40	2.48
DRE3	6.61	1.39	CRE3	6.66	2.37
DRE4	9.04	1.43	CRE4	7.81	2.26

Supplementary Table 4 - Averaged errors of the estimated model parameters for the model of MAP kinase pathway.

Criterion	Discrete criterion		Criterion	Continuous criterion	
	Mean error	STD		Mean error	STD
DRE1	349.40	84.86	CRE1	<u>225.97</u>	59.16
DRE2	299.63	106.91	CRE2	<u>261.50</u>	<u>27.66</u>
DRE3	280.99	69.26	CRE3	262.03	66.92
DRE4	331.85	112.98	CRE4	313.86	58.50

Supplementary Table 5 - Averaged errors of the estimated factors ($K_{mi} = (d_i + k_i)/a_i$) for the model of MAP kinase pathway.

Criterion	Discrete criterion			Continuous criterion	
	Mean error	STD error		Mean error	STD error
DRE1	39.07	24.77	CRE1	<u>16.03</u>	<u>5.45</u>
DRE2	255.40	709.32	CRE2	79.13	74.20
DRE3	183.70	207.37	CRE3	155.64	176.89
DRE4	23.35	8.30	CRE4	59.01	56.01

Supplementary Table 6 - Robustness property of the estimated parameters for the model of the ERK kinase activation (4) (FV: fitness value)

Criterion	Discrete criterion			Continuous criterion	
	Mean-FV	STD-FV	Criterion	Mean-FV	STD-FV
DAE1	0.059	0.037	CAE1	0.058	0.036
DAE2	0.059	0.036	CAE2	0.059	0.036
DAE 3	0.059	0.036	CAE3	0.052	0.030
DAE 4	0.061	0.037	CAE4	0.051	0.029
DRE 1	0.059	0.036	CRE1	0.060	0.037
DRE 2	0.12	0.086	CRE2	0.059	0.036
DRE 3	0.11	0.082	CRE3	0.061	0.037
DRE 4	0.11	0.081	CRE4	0.059	0.036

Supplementary Table 7 - Robustness property of the estimated parameters for the model of the G1/S transition module.

Criterion	Discrete criterion			Continuous criterion	
	Mean-FV	STD-FV	Criterion	Mean-FV	STD-FV
DAE1	0.607	0.413	CAE1	0.530	0.351
DAE2	0.506	0.336	CAE2	0.507	0.335
DAE3	0.499	0.326	CAE3	0.494	0.323
DAE4	0.504	0.329	CAE4	0.513	0.331
DRE1	0.474	0.316	CRE1	0.513	0.346
DRE2	0.578	0.363	CRE2	0.762	0.466
DRE3	0.666	0.429	CRE3	0.718	0.456
DRE4	0.716	0.425	CRE4	0.677	0.408

Supplementary Table 8 - Robustness property of the estimated parameters for the model of the MAP kinase pathway.

The activated ERK kinase activity (namely ERKpp) in the MAP kinase pathway at 5min was used to calculate the robustness property.

Criterion	Discrete criterion			Continuous criterion	
	Mean-FV	STD-FV	Criterion	Mean-FV	STD-FV
DRE1	2328.2	1.416	CRE1	43.55	1.080
DRE2	240.8	0.832	CRE2	136.37	1.909
DRE3	1076.1	0.139	CRE3	313.94	0.250
DRE4	521.2	0.724	CRE4	581.57	0.064

Supplementary Figure 1 - Simulations of the ERK kinase activation module.

One simulation of system (21) is obtained using the standard rate constants in Eq. (22). Experimental data in Ref [34] are presented by red dots.

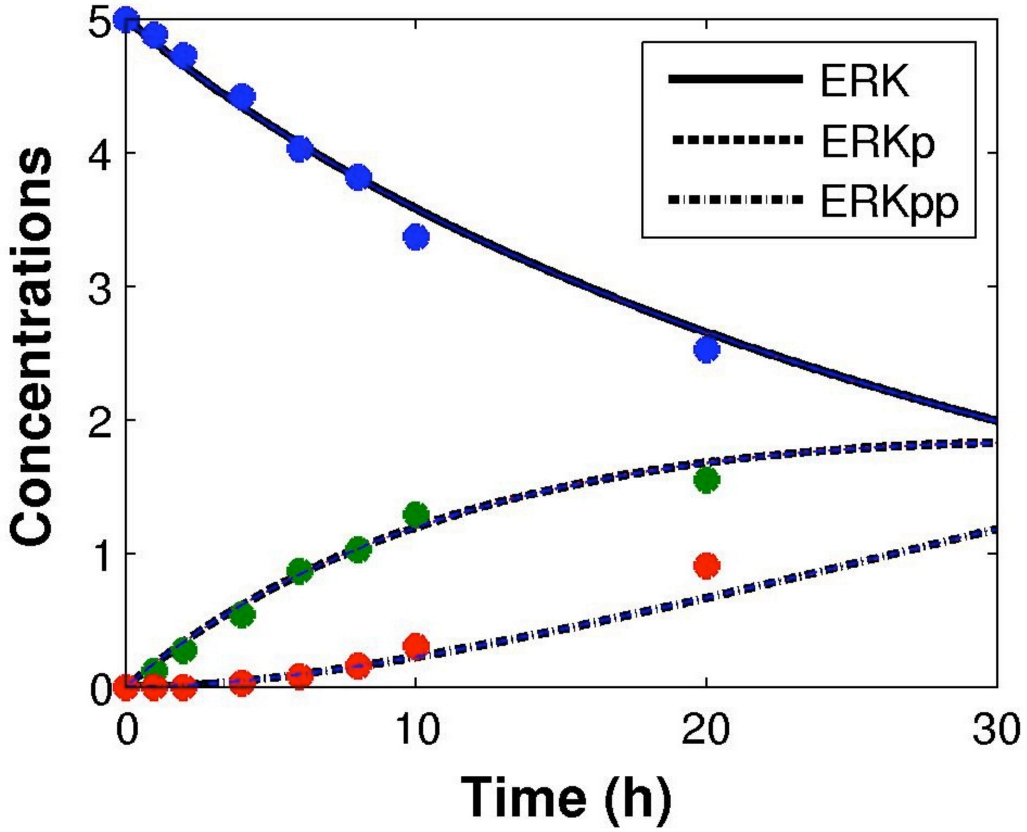

Supplementary Figure 2 - Estimated rate constants of the ERK kinase activation module.

Each estimated parameter was scaled by the corresponding standard parameter in Eq. (21); and the standard value of each parameter is represented by unit one (the horizontal line) in each sub-figure. Model indexes 1~8 are for the discrete criteria: 1: DAE1, 2: DAE2, 3: DAE3, 4: DAE4, 5: DRE1, 6: DRE2, 7: DRE3, 8: DRE4. Model indexes 9~16 are for the continuous criteria: 9: CAE1, 10: CAE2, 11: CAE3, 12: CAE4, 13: CRE1, 14: CRE2, 15: CRE3, 16: CRE4.

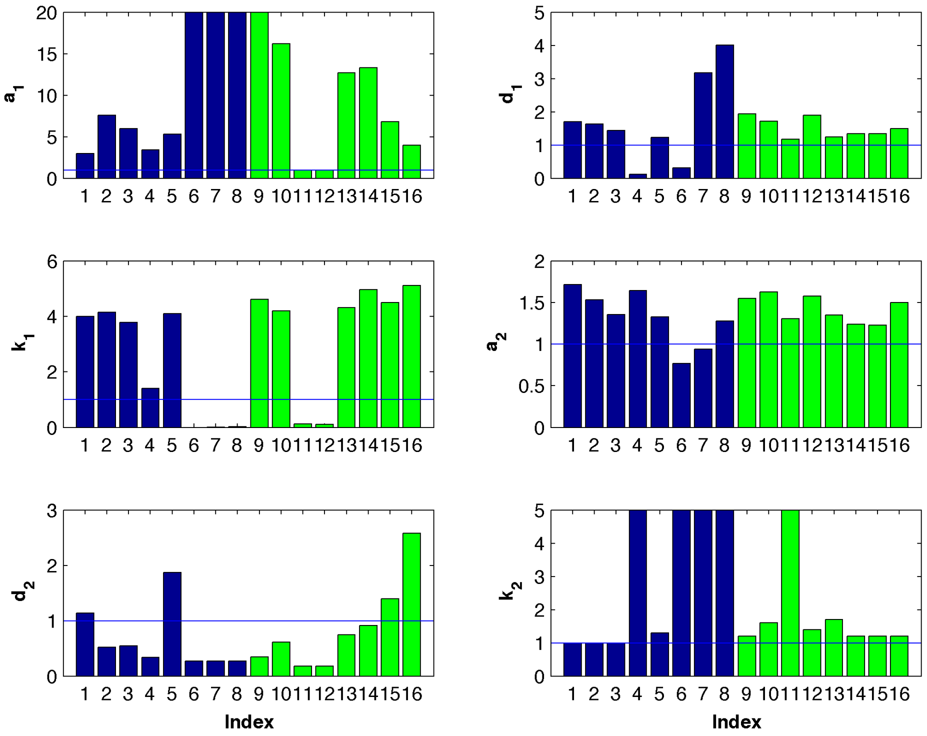

Supplementary Figure 3 - Simulations of the G1/S transition module.

(Solid-line: [pRB], dash-line: [E2F1], star: discrete simulation used as experimental data).

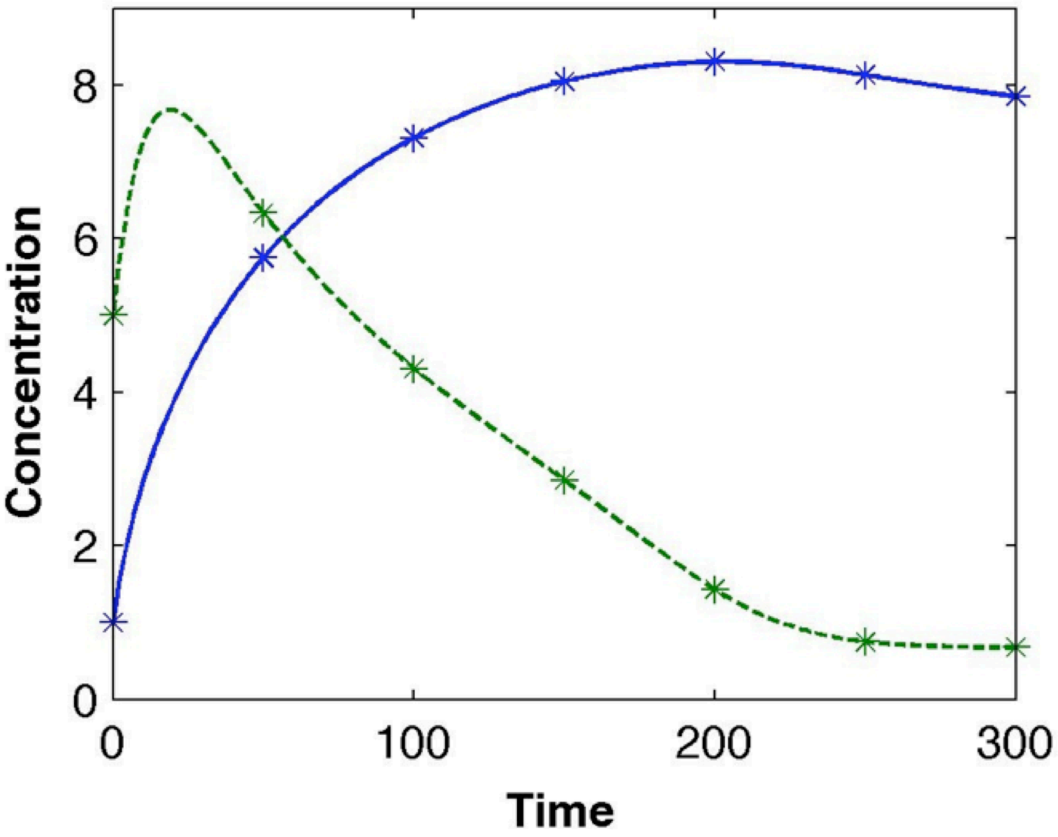

Supplementary Figure 4 – Simulation of the MAP kinase pathway

(Solid-line: kinase activities at different locations; star: extended proteomic data using a linear interpolation).

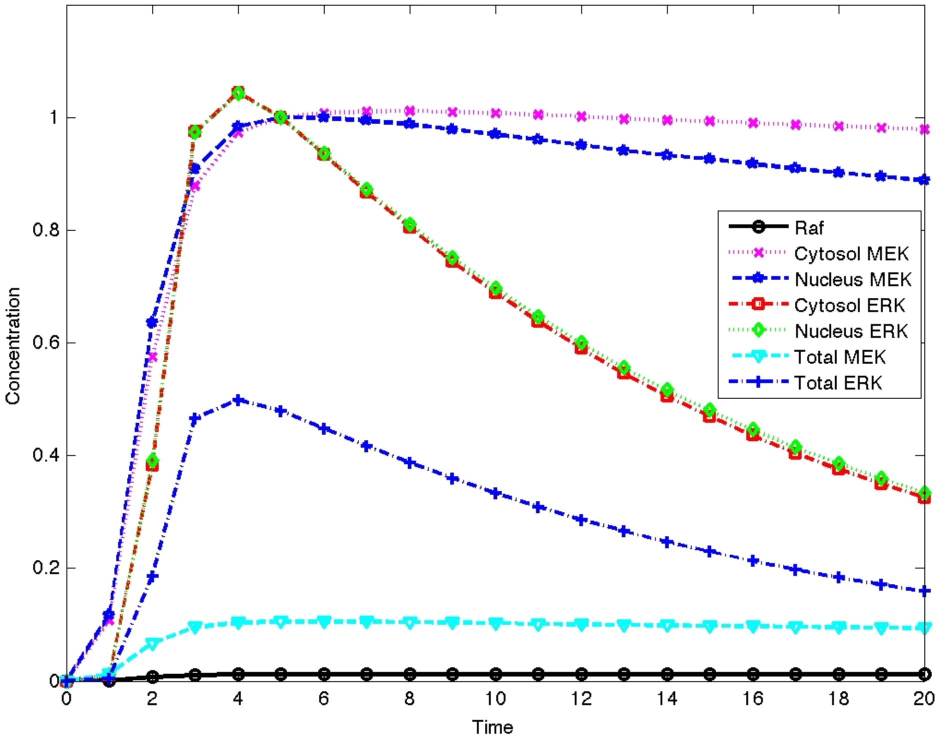

Reference

1. Tian T, Song J: **Mathematical modelling of the MAP kinase pathway using proteomic datasets**. *PLoS One* 2012, **7**(8):e42230.
